# TLR7 and TLR9 have overlapping but distinct roles in systemic lupus erythematosus and Sjögren’s disease

**DOI:** 10.3389/fimmu.2026.1802432

**Published:** 2026-04-23

**Authors:** Sheta Biswas, Jill M. Kramer

**Affiliations:** 1 Department of Oral Biology, School of Dental Medicine, The University at Buffalo, State University of New York, Buffalo, NY, United States; 2 Department of Biochemistry and Molecular Biology, Noakhali Science and Technology University, Noakhali, Bangladesh

**Keywords:** age-associated B cell, autoantibodies, plasmacytoid dendritic cell (pDC), salivary gland, XIST

## Abstract

Endosomal toll-like receptors (TLRs), such as TLR7 and TLR9, are key mediators of autoimmunity. Systemic lupus erythematosus (SLE or lupus) and SjD are distinct diseases that are both characterized by heightened activation of TLR7 and TLR9 signaling networks that serve as potent modulators of chronic inflammation. This review will provide an overview of the role of these receptors in lupus and SjD, with a focus on recent mechanistic insights in the field. We will compare and contrast the roles of TLR7 and TLR9 in lupus and SjD, with a focus on the importance of B cell activation in disease. Moreover, we will discuss differences observed in sex-biased organ-specific disease manifestations. Finally, we will review current and emerging therapies that target endosomal TLR pathways and discuss their utility for treatment of SLE and SjD.

## 1 Introduction

Autoimmune disease is characterized by the loss of immunological self-tolerance, and encompasses a broad spectrum of clinical manifestations (1). About 8% of Americans are diagnosed with autoimmunity (2). Systemic lupus erythematosus (SLE or lupus) is an autoimmune disease that causes chronic inflammation and immune-mediated damage in multiple organ systems including kidneys, skin, and lungs (3, 4). Approximately 3.4 million individuals worldwide are affected by SLE, and 400,000 new cases are diagnosed annually (3). SLE is most prevalent among women of reproductive age, with a notably higher incidence observed in those of African origin (3, 5). Sjögren’s Disease (SjD) also has a striking female predilection and is among the most prevalent autoimmune diseases in the US (6, 7). Globally, it is estimated that 0.01 – 0.05% of the population is affected by SjD (8, 9). SjD is characterized by chronic inflammation in the exocrine tissues, and accompanying loss of function of salivary and lacrimal glands (10). In addition to exocrine dysfunction, it is estimated that 30 - 50% of patients with SjD show extra-glandular manifestations such as pulmonary, neurological, renal, and articular involvement (10, 11). A comparison of the organ-specific disease manifestations in SLE and SjD is provided in **Table 1** (4, 10, 12–21).

**Table 1:** Organ-specific disease manifestations in SLE and SjD.

SLE		SjD	
Organ-specific manifestations	Prevalence	Organ-specific manifestations	Prevalence
• Mucosal: Oral ulcers	30%	• Exocrine/Salivary: Loss of saliva production	60 - 70%
		• Exocrine/Lacrimal: Diminished tear production (Schirmer’s test)	43%
• Renal: Lupus nephritis (asymptomatic proteinuria, nephrotic syndrome, acute kidney injury)	40%	• Renal: Tubulointerstitial nephritis or cryoglobulinemia-associated glomerulonephritis	5 - 50%
• Cardiopulmonary: Pericarditis, pleuritis	3 – 25%	• Pulmonary: Chronic bronchitis, bronchiolitis or interstitial lung disease	10 - 20%
Hematopoietic: • Autoantibodies (ANA > 1:80, anti-dsDNA, anti-nucleosome, anti-histone, anti-Ro, anti-Smith, anti-RNP) • Thrombocytopenia • Hemolytic anemia • Leukopenia	30 – 100% 9% 4 - 12% 22 – 42%	Hematopoietic: • Hyper-gammaglobulinemia • Autoantibodies (ANA > 1:320, RF, anti- Ro, anti-La) • Low complement C4 • Anemia	39 – 55% 49 – 76% 18% 20%
Neuropsychiatric (CNS and PNS): • Headache • Mood disorders • Cognitive impairment • Seizures • Psychosis • Poly-neuropathy, peripheral neuropathy	11 – 96%	• CNS: Cerebral vasculitis, transverse myelitis or demyelinating lesions, hemiparesis, paraparesis, dysphonia • PNS: Pure sensory neuropathy, sensimotor neuropathy ataxic gangliopathy or vasculitis (mononeuritis multiplex)	2 – 10% 6 – 16%
• Muscular: Myositis (diffuse myalgia and muscle tenderness)	4%	• Muscular: Myositis with pain and weakness	1 - 2%
Articular: • Non-deforming non-erosive arthritis • Deforming arthropathy • Erosive arthropathy	69%		
• Gastrointestinal: Protein-losing enteropathy, hepatitis, pancreatitis, intestinal pseudo-obstruction	< 10%	• Articular: Arthralgias with morning stiffness or synovitis	38 – 86%
• Cutaneous: Malar rash, photosensitivity, Raynaud phenomenon, non-scarring alopecia	10 – 70%	• Cutaneous: Purpura, vasculitis, Raynaud phenomenon	10 - 30%

Despite being distinct diseases, SLE and SjD have common epidemiologic and molecular features. Both SLE and SjD are among the most female-biased autoimmune diseases (6). Molecular studies also document similarities between the two diseases, as the majority of patients display an interferon (IFN) gene signature (22). Moreover, SLE and SjD patients have overlapping autoantibody profiles, including those directed against nucleic acids (23). In agreement with their shared clinical attributes, SLE and SjD are driven, at least in part, by overlapping signaling networks. Indeed, many studies demonstrate that aberrant activation of Toll-like receptors (TLRs) contributes to both diseases (24, 25).

TLRs are germline-encoded pattern recognition receptors that recognize conserved pathogen-associated molecular patterns (PAMPs) and endogenous danger signals, referred to as damage-associated molecular patterns (DAMPs) (26). TLRs are type I transmembrane proteins that consist of an amino-terminal ectodomain, a transmembrane domain, and a carboxy-terminal Toll interleukin-1 receptor (IL-1R) TIR domain within the cytoplasmic tail (1). TLRs are broadly expressed by many immune populations including B cells, plasmacytoid dendritic cell (pDCs), monocytes, and macrophages as well as non-immune cells, such as epithelium (27–29). Since TLRs can recognize host-derived moieties, activation of these receptors has been identified as a key pathologic mechanism in autoimmunity in both mice and humans (30).

Indeed, studies in mice demonstrate that activation of TLR7 and TLR9 mediates lupus pathogenesis in several different models of disease (31–38). Corroborative findings in SLE patients have identified SNPs in *TLR7* or *TLR9* as contributing risk factors for SLE development (39–41), and gain of function (GOF) mutations are identified in *TLR7* and in downstream signaling molecules employed by endosomal TLRs in patients with SLE (42–48). While the roles of TLR7 and TLR9 are well established in lupus, these receptors are not as well understood in the context of SjD. There is evidence that TLR7 and TLR9 show altered expression and activation in patients and mouse models of SjD (49–54), but there are relatively few studies that have evaluated endosomal TLRs in this disease. Recent work performed in mice, however, indicate key roles for both TLR7 and TLR9 in SjD (52, 55–61), and these findings are supported by those in SjD patients (50, 53, 54, 62–64). Of note, while endosomal TLRs show many similarities in the way in which they modulate disease in the context of SLE and SjD, there are also distinct differences observed.

In this review, we will summarize the contributions of TLR7 and TLR9 signaling to SLE, and we will compare these findings to those in SjD models and patients. We will discuss TLR7 and TLR9 in the context of lupus, with particular emphasis on their opposing roles in disease and the sex-biased effects observed. We will then focus on the role of these receptors in SjD and compare and contrast the roles of these receptors in lupus and SjD. Last, we review current and emerging therapies that target endosomal TLRs pathways and discuss their utility for treatment of SLE and SjD.

## 2 TLR7 and TLR9 signaling pathways mediate lupus

### 2.1 TLR7 activation drives SLE pathogenesis

TLR7 is a potent driver of autoimmunity, and its role in disease in particularly well-established in lupus. Seminal studies that revealed a role for TLR7 in lupus were carried out in the recombinant BXSB mouse strain, as robust lupus-like disease was observed in male mice. Subsequent work revealed that disease was driven by genes encoded by the Y-linked autoimmune accelerating (*Yaa*) locus, and the *Yaa* phenotype was found to be conferred by TLR7 overexpression (65). Numerous corroborative studies in this and other mouse models have established that TLR7 is a prime driver of lupus (66–75).

Findings in mice are well supported by those in humans. Indeed, genetic studies in SLE patients demonstrate both copy number variations and single-gene polymorphisms (SNPs) within the *TLR7* locus contribute to SLE susceptibility (39–41). Of note, TLR7 is located on the X-chromosome, and improper X-chromosome inactivation mediated by *XIST* is documented in B cells from female SLE patients, leading to TLR7 overexpression (76, 77). Further evidence for the importance of TLR7 activation in disease is provided by studies demonstrating monogenetic GOF mutations in *TLR7* and downstream signaling intermediates in SLE patients (42–48). A summary of these mutations is provided in **Table 2**. Indeed, studies of a female with lupus identified a mutation in *TLR7* that led to increased affinity for select TLR7 ligands (42). When this same *TLR7* mutation was expressed in mice, B cell receptor (BCR)-activated B cells showed enhanced survival, and expansion of germinal center (GC) and age-associated B cells (ABCs) was noted, two B cell subsets that mediate lupus pathology (42). Altogether, data from mice and humans demonstrate that TLR7 activation is sufficient to drive lupus pathogenesis.

**Table 2:** GOF mutations in TLR7 signaling pathways in SLE patients.

Gene	Variant	Mechanism	Clinical findings	Murine phenotype	Ref.
*TLR7*	Y264H	Increased affinity for guanosine and 2’,3’ cGMP Enhanced NFκB activation following stimulation with TLR7 agonists	Childhood SLE	Autoimmunity Expansion of ABCs and GC B cells Aberrant B cell survival	(42)
	F507L	Increased sensing of 2’,3’ cGMP Enhanced NFκB activation following stimulation with TLR7 agonists	Mother has SLE Daughter has neuromyelitis optica	ND	(42)
	P267L	TLR7 protein expression increased in B cells, monocytes and DCs Enhanced NFκB activation following stimulation with TLR7 agonists	Childhood SLE Inflammatory vasculitis Sudden-onset status epilepticus	ND	(43)
	F506S	Enhanced NFκB activation following stimulation with TLR7 agonists Increased affinity for ssRNA	Early-onset SLE	ND	(44)
*UNC93B1*	G325C	Enhanced NFκB activation following stimulation with TLR7 and TLR8 agonists	Childhood SLE	ND	(45)
	I317M	Enhanced NFκB activation following stimulation with TLR7 agonist	Childhood SLE	ND	(45)
	R336L and E92G	Altered steady state of TLR7 activation toward higher responsiveness	Childhood SLE	ND	(47)
	R336C	Enhanced interaction with Syntenin-1 Disruption in regulatory steps downstream of UNC93B1 ubiquitylation and Syntenin-1 binding	Childhood onset cutaneous tumid and chillblain lupus Inflammatory arthritis	Systemic autoimmunity Splenomegaly Expansion of ABCs, plasma cells, and monocytes Elevated chemokines and ANAs Glomerulonephritis Increased responsiveness to TLR7 ligation	(46)
	T93I	Reduced TLR7 binding May enable TLR7 to bind ligand more easily and dimerize while still associated with UNC93B1	Early-childhood onset cutaneous tumid lupus	Splenomegaly Enhanced GC B cells, ABCs, plasma cells, Tfh cells, and monocytes Heightened responses to TLR7 ligands	(46)
*PASCIN1*	Q59K	Enhanced TLR7 signaling Increased binding to N-WASP protein and decreased binding to TRAF4, resulting in unrestrained TRAF6-mediated activation of type I IFN	Childhood SLE	Heterozygous animals appeared healthy Enhanced TLR7 expression in B cells Increased TRAIL+ B cells in males Elevated anti-chromatin antibodies in males	(48)

cGMP, cyclic guanosine monophosphate; ND, Not determined.

### 2.2 TLR9 plays a predominately protective role in SLE

In contrast to TLR7, abundant evidence indicates that TLR9 is protective in the context of lupus (32, 78–84), as TLR9-deficient lupus mice developed severe lupus nephritis and decreased survival (27, 32, 70, 79). Additionally, lupus-prone MRL/lpr mice lacking TLR9 systemically showed higher total serum IgG and autoantibody levels (33, 80). TLR9-deficient mice also displayed proteinuria, dermatitis, and lymphadenopathy that was accompanied by increased mortality (84).

While genetic studies in patients with SLE have failed to link polymorphisms in *TLR9* consistently with disease (39), numerous studies suggest that TLR9 expression is enhanced in SLE patients. TLR9 expression is reported to be elevated in PBMCs, and in B cells and pDCs in the context of lupus (85–92). Moreover, B cell TLR9 was enhanced in SLE patients compared to those from healthy controls and TLR9 expression positively correlated with serum IL-6 and anti-dsDNA antibodies (39, 91).

At first glance, such findings in patients seem contradictory to those in mice, because elevated TLR9 would be expected to confer an immunoregulatory effect that would lead to suppression of autoimmunity. Several studies offer a possible explanation for these seemingly disparate observations. Although TLR9 expression was elevated, stimulation of TLR9 in immune cells from SLE patients resulted in attenuated inflammatory responses when compared to those from healthy controls (89, 93–97). Indeed, B cells and monocytes derived from SLE patients with severe disease exhibited profound TLR9 hyporesponsiveness, as evidenced by diminished activation, proliferation, and cytokine secretion (95–97). Taken together, data from both mice and humans indicate that TLR9 agonism protects against disease in the context of lupus in both mice and humans, although further studies are needed to understand the mechanistic underpinnings of these observations, as discussed in detail below.

### 2.3 TLR7 and TLR9 activation in B cells contributes to distinct manifestations of lupus

TLR7 and TLR9 are highly expressed in B cells in mice and humans (27, 98). Numerous studies demonstrate that B cells in lupus patients exhibit aberrant responses to self-antigens, resulting in the formation of excessive autoantibodies (99, 100). In SLE, defective clearance of apoptotic cells leads to the persistence of self-antigens, including DNA, RNA, and nuclear proteins (101, 102). This results in the generation of DAMPs that may serve as ligands for endosomal TLRs (30).

Indeed, nucleic acid-containing apoptotic debris triggers the activation of autoreactive B cells through co-engagement of the BCR and TLRs, and this mediates the production of antinuclear autoantibodies (ANAs) in the context of lupus (103, 104). TLR7 preferentially senses uridine-rich RNA including small nuclear RNAs associated with small nuclear ribonucleoproteins (snRNPs) (105–107). In addition, chromatin-IgG complexes mediate the activation of autoreactive B cells through ligation of the BCR and TLR9 (38). Both TLR7 and TLR9 signals differentially shape the B cell repertoire in the context of lupus, as TLR7 activation facilitates the generation of antibodies targeting RNA-associated antigens and TLR9 preferentially drives the production of antibodies against dsDNA and chromatin (32, 70, 80, 108). A summary of the role in B cell TLR7 and TLR9 in lupus is provided in the following section.

### 2.4 TLR7 and TLR9 mediate expansion of B cell subsets integral to lupus pathogenesis

#### 2.4.1 Lupus mouse models reveal the importance of B cell-intrinsic TLR7 and TLR9 in disease

Elegant studies have established the importance of B cell-intrinsic TLR7 expression in the context of lupus, as ablation of TLR7 in the B cell compartment of lupus mice resulted in reduced autoantibodies against RNA-associated autoantigens and diminished glomerulonephritis and proteinuria (27, 32). Additional work provided corroborative evidence that B cell-intrinsic TLR7 mediates lupus, as ablation of the gene that encodes NOX2 (*Cybb*) specifically in B cells in a lupus prone model resulted in robust interstitial and glomerulonephritis and elevated anti-Sm and anti-RNA autoantibodies (109). Strikingly, these were significantly reduced when both *Cybb* and *TLR7* were deleted in the B cell compartment, indicating that the disease observed in the absence of NOX2 was dependent on B cell TLR7 expression (109).

Moreover, a seminal study detailed the importance of B cell-intrinsic TLR7 in the formation of spontaneous GCs in the context of lupus (110). Spontaneous GCs are a hallmark of several lupus models and are thought to be important early mediators of loss of tolerance (111). Work using bone marrow chimeras that received bone marrow cells from either TLR7- or TLR9-deficient *B6.Sle1b* lupus-prone mice established that TLR7 expressing B cells were required for the generation of spontaneous GCs, while TLR9 in the B cell compartment was dispensable (110). These data, in combination with additional studies, established that expression of at least one copy of TLR7 in B cells was necessary for the formation of spontaneous GCs in the context of lupus (110, 112–114).

Additional work has examined the role of B cell-intrinsic TLR9 in the context of lupus in depth. Indeed, autoimmune-prone mice that lacked expression of TLR9 in B cells had enhanced glomerulonephritis and effector memory T cells (32). Corroborative work examined MRL/lpr mice in which TLR9 was either ablated in the B cell compartment or was overexpressed only in B cells (35). B cell-intrinsic overexpression of TLR9 diminished proteinuria, ameliorated lupus nephritis, and decreased serum titers of anti-RNA IgG (35), while ablation of B cell TLR9 led to exacerbation of disease (35). Strikingly, this work revealed that significant protection from lupus was conferred by TLR9 signaling in the B cell compartment.

The importance of endosomal TLR signaling in B cells was further clarified in a study in which TLR7 was ablated only in the B cell compartment in lupus prone mice that also lacked systemic expression of TLR9 (27). This study revealed that TLR7 activation in B cells contributed significantly to disease and suggested that blocking B cell TLR7 accompanied by concomitant TLR9 agonism may be an effective therapeutic approach for lupus (27). Taken together, these studies demonstrate that B cell TLR7 and TLR9 are required for robust development of lupus.

Recent work has focused on understanding the contribution of a B cell subset to lupus pathogenesis that is preferentially activated by endosomal TLRs, termed ABCs. It is important to note that TLR7 and TLR9 drive the differentiation of ABCs, which are class-switched, antigen-specific memory B cells (115, 116). In the presence of IFNγ and IL-21, treatment of naïve B cells with either a TLR7 or TLR9 agonist leads to the generation ABCs in both autoimmune-prone humans and animal models (117, 118). TLR7 is crucial for driving expansion of this population with age in healthy mice (115, 119), and ABCs are diminished significantly in TLR7-deficient lupus-prone mice (120). TLR9 agonism also promotes the differentiation and proliferation of ABCs (116, 119).

Elegant work in the DEF6/SWAP70 double knockout (DKO) lupus model established the importance of TLR7 expression in driving the expansion of ABCs in lupus. This model was generated by ablating the SWEF proteins DEF6 and SWAP-70 systemically. These proteins belong to a family of small guanine exchange factors, and polymorphisms in either *DEF6* or *SWAP-70* are associated with increased risk for development of autoimmunity (121–123). The DKO model demonstrates a lupus-like disease that primarily affects females and is characterized by expansion of ABCs (124–127). DKO female mice show increased of splenic B cell populations, elevated autoantibodies, and heightened lung inflammation as compared to strain-matched males (128).

Importantly, when TLR7 was overexpressed in DKO males, (termed Yaa-DKO mice), this strain exhibited ABC expansion, along with increased numbers of GC B cells and plasmablasts/plasma cells. Robust pulmonary pathology and increased mortality was also observed in the Yaa-DKO males as compared to DKO males and females. Significantly, increased percentages of ABCs were observed in the Yaa-DKO males relative to the other strains, indicating the importance of TLR7 agonism in driving ABC expansion and subsequent disease in the context of lupus (128).

Of note, recent work performed in a lupus mouse model using adoptively transferred B cells revealed that naïve-derived CD21^lo^ cells are precursors of extrafollicular antibody secreting cells, and this population requires TLR7 activation for early escape of peripheral tolerance (129). Moreover, repertoire analysis showed that in CD21^lo^ B cells, the BCR evolves toward self-reactivity (129). Altogether, these data indicate that ABCs have clinical relevance in the context of SLE, and TLR7 agonism mediates their expansion in disease.

#### 2.4.2 B cells derived from SLE patients show aberrant endosomal TLR function

In SLE patients, numerous defects in TLR7 and TLR9 signaling in B cells are documented (98, 130, 131). In particular, endosomal TLR activation in GC B cells, ABCs, and extrafollicular B cells is shown to mediate disease.

ABCs are particularly sensitive to TLR7 ligation, and expansion of these cells, also referred to as double negative (DN) or atypical B cells in humans, is well documented in SLE patients (132, 133). Of note, the term “atypical” is imprecise and it is suggested that this term no longer be used to describe this subset (133). DN B cells, characterized as IgD-,CD27-,CXCR5-,CD11c+, arise through extrafollicular differentiation and are enriched in TLR7 expression and also display hyperresponsiveness to TLR7 ligation (132). This population was expanded in African American patients with active disease who had nephritis, and anti-Sm and anti-RNA autoantibodies (132). Similar to findings in mice, stimulation of naïve B cells with IL-21 and IFNγ along with TLR7 agonism drove their differentiation into DN B cells and plasma cells (132). Corroborative work revealed that among patients with SLE, those with lupus nephritis had elevated DN B cells in the periphery, and patients who responded well to treatment, as measured by reduced urinary protein excretion, had diminished percentages of DN B cells (134).

Although TLR7 is studied much more extensively than TLR9, TLR9 activation in B cells also mediates pathology in lupus patients. Lupus B cells display impaired responses to TLR9 agonism (93). Since TLR9 signaling is crucial for the establishment of central B cell tolerance, both in mice and humans (135–137), this hyporesponsiveness could be an important mechanism leading to loss of self-tolerance in SLE. It is important to point out that CXCL4 (also referred to as platelet factor-4 or PF-4) may contribute to this TLR9-mediated loss of tolerance, as CXCL4 inhibits TLR9 activation in B cells by sequestering ligands from the endosomal compartment and thereby prevents their engagement with TLR9 (136). This finding is clinically significant because in many autoimmune diseases, including SLE, patients exhibit elevated CXCL4 levels that may contribute to diminished TLR9-dependent B cell tolerance (136, 138, 139).

### 2.5 TLR9 governs TLR7 activation in lupus

Studies in which both TLR7 and TLR9 were ablated systemically in lupus models have led to important insights regarding the regulatory relationship between these receptors. Since the contributions of TLR7 and TLR9 signaling appear to be paradoxical in lupus (32, 70), numerous elegant experiments have provided a mechanistic framework that has enhanced our understanding of the crosstalk between these receptors in disease. A brief summary of these findings is provided below.

In a seminal study that explored interactions between TLR7 and TLR9, MRL/lpr mice were generated that lacked systemic expression of both receptors, either alone or in combination (33). This work revealed that disease manifestations in TLR9-deficient mice were mediated by heightened TLR7 activation, as MRL/lpr mice lacking both TLR7 and TLR9 were broadly protected from lupus. Moreover, both MRL/lpr*^Tlr7y/-^* mice and MRL/lpr mice lacking both TLR7 and TLR9 exhibited significant reduction in disease compared to the strain matched TLR9-deficient mice (33). These findings indicated that the pathogenic effects of TLR7 dominate over the protective influence of TLR9 and suggested that these receptors may regulate each other in the context of lupus.

Additional work focused on the roles of TLR7 and TLR9 in B cells specifically (140). Indeed, this seminal study analyzed B cells derived from lupus-prone mice that lacked TLR7 or TLR9 alone or both receptors in combination. Adoptive transfer experiments were conducted in which B cells were transferred either from mice lacking *TLR7*, *TLR9*, or from mice lacking both receptors in combination into the TLR-sufficient parental strain. The recipient mice were then given dual BCR/TLR7 and BCR/TLR9 agonists. This study revealed that *TLR9-/-* B cells showed enhanced differentiation into IgG+ antibody forming cells as compared to the *TLR7-/-* B cells or the B cells that lacked both TLRs (140). Of note, TLR7 expression was similar between TLR9-deficient B cells and those derived from the parental strain in the presence or absence of TLR7 stimulation, so the underlying mechanisms for this observation remained poorly understood (140). Nonetheless, this work provided compelling evidence that TLR9 governs TLR7 activation in B cells.

Further studies to explore this regulatory relationship have focused on UNC93B1, a protein that regulates endosomal TLR trafficking and is required for activation of TLR7 and TLR9. SLE patients show GOF mutations in *UNC93B1* that drive disease (45–47). Of direct relevance to lupus, studies in B cells reveal that TLR7 and TLR9 compete for UNC93B1, with TLR9 dominating due to its higher binding affinity (141). Mutations in *UNC93B1* are identified in SLE patients that led to TLR7, but not TLR9, hyperresponsiveness and constitutive type I IFN signaling (47). Further work established that the expression of TLR7 was governed by UNC93B1 through its interaction with syntenin-1, as a mutation in *UNC93B1* that impaired its interaction with syntenin-1 led to systemic inflammation in mice, recapitulating the disease phenotype induced by TLR7-overexpression (142). Altogether, these data indicate that UNC93B1 plays a crucial role in controlling TLR7 and TLR9 activation, and functions to prevent excessive TLR7 signaling that is characteristic of lupus.

To explore the regulation of TLR7 by TLR9 in greater depth, studies were carried out using both healthy and lupus-prone mice with *TLR9* point mutations that either hindered ligand binding or interfered with MyD88-dependent signaling. Through elegant adoptive transfer experiments, this work established that TLR9 can initiate two distinct signaling pathways in B cells: one that is MyD88-dependent and pro-inflammatory in nature and a second ligand-dependent MyD88-independent regulatory pathway (34). This work demonstrated that TLR9 has both ligand- and MyD88-independent regulatory roles, and these data indicate that TLR9 does not directly regulate TLR7 in B cells in the context of lupus.

**Figure 1:**
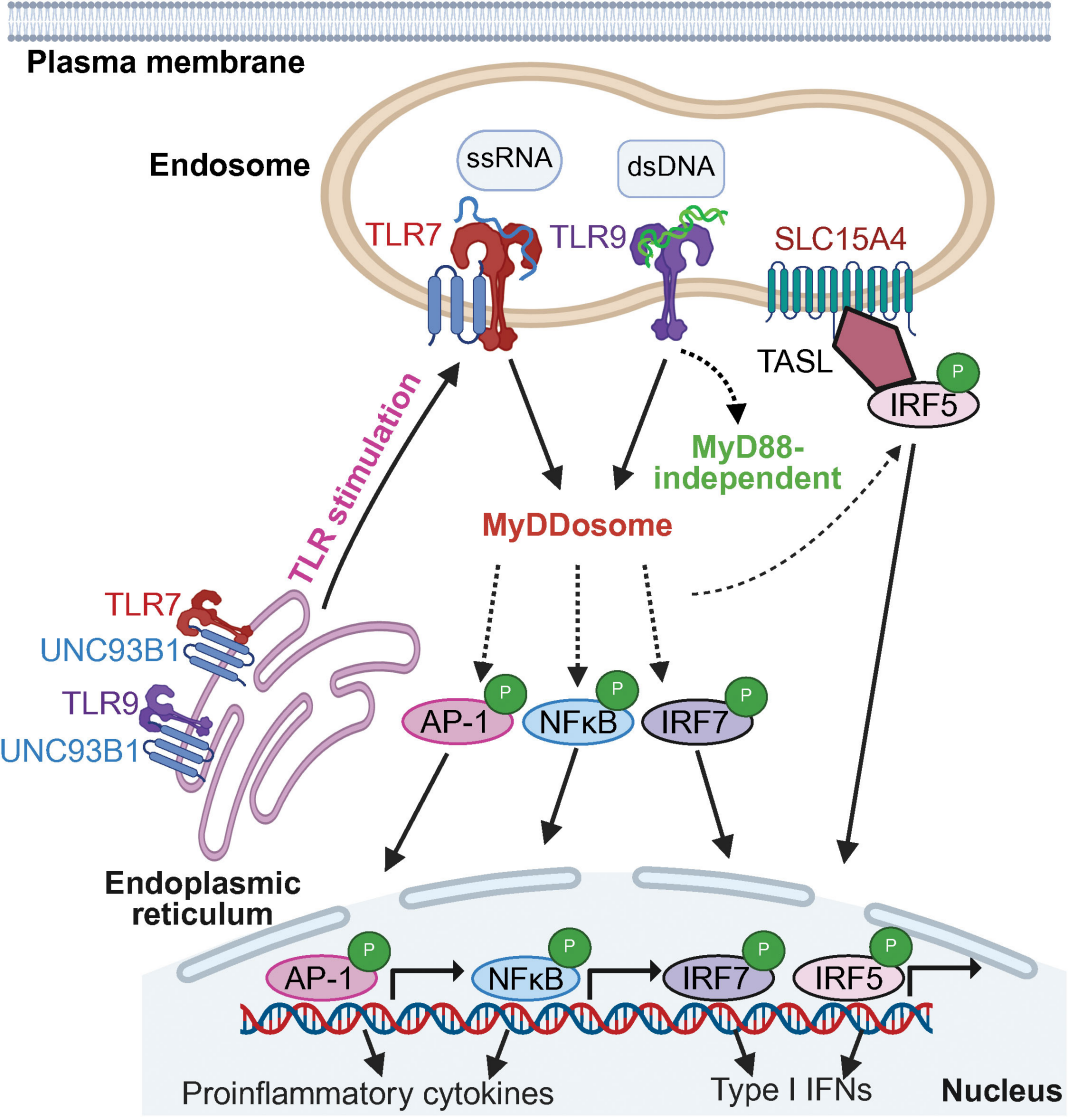
Summary of TLR7 and TLR9 signaling networks. Signaling intermediates used by TLR7 and TLR9 are shown. Figure is adapted from (34, 142, 191, 215, 216).

This work then led the authors to hypothesize that the dichotomous effects of TLR7 and TLR9 may be mediated by their respective TIR domains, since these domains are only about 46% homologous between the two receptors and thus could confer different functionality (143). To investigate this, they generated mice in which the TIR domains of each receptor were swapped, such that the TLR9 TIR domain was replaced with that of TLR7, and vice versa. The resultant mice expressed TLR7 that contained the TLR9 TIR domain (termed TLR779) and TLR9 with a TLR7 TIR domain (termed TLR997). Interestingly, TLR779 lupus-prone mice that expressed the TLR9 TIR domain within TLR7 had markedly attenuated disease, whereas the TLR997 MRL/lpr mice had exacerbated disease relative to the MRL/lpr parental strain. This study demonstrated that each TIR domain serves as a critical determinant of the opposing effects of TLR7 and TLR9 signaling in lupus (143). Collectively, these findings provide a mechanistic understanding of the way in which TLR7 and TLR9 control opposing signals in the context of lupus, and further studies will be insightful to identify specific TIR domain-mediated outcomes in this disease. A summary of key signaling pathways utilized by TLR7 and TLR9 is provided in **Figure 1**.

In summary, there is abundant data derived from both mouse models and SLE patients that demonstrate a critical role for endosomal TLRs in controlling organ-specific disease manifestations. Studies in lupus models have been particularly valuable in establishing the importance of B cell-intrinsic TLR7 and TLR9 signaling cascades to disease. Corroborative studies in humans provide compelling evidence that support the role of TLR7-mediated ABC expansion. Finally, elegant work from several groups highlights the important regulatory relationship between TLR7 and TLR9 and demonstrates that this is dysregulated in the context of SLE. Thus, activation of these signaling cascades is a key driver of disease in both mice and humans.

## 3 TLR7 and TLR9 mediate SjD pathogenesis

### 3.1 TLR7 expression and function are altered in SjD models and patients

Several groups have investigated the role of TLR7 in SjD using mouse models, and this work has established an integral role for TLR7 in disease (52, 57–59, 61). A summary of these findings is provided in **Table 3**.

**Table 3:** Overview of TLR7-mediated disease in SjD mouse models.

Model/ Ref.	Description	Phenotype
TLR7/8 ko mice (C57BL/6)/ (58)	Systemic ablation of TLR7 and TLR8 TLR7/8 ko mice develop lupus and SjD	*Compared to TLR8 ko age-/ sex-matched mice:* Females: IgM and IgG deposition absent in SMGs Ectopic lymphoid structures (B/T cell aggregates) abrogated, diminished cytokines in SMG Lung infiltration abrogated Diminished anti-Ro and anti-La antibodies in sera
NOD.ShiLt/J*^Tlr7-/-^*/ (59)	Systemic ablation of TLR7 NOD.ShiLt/J mice develop Type 1 Diabetes (T1D) and SjD	*Compared to NOD.ShiLt/J age-/ sex-matched mice:* Females: No change in sialadenitis or T1D Males: Enhanced sialadenitis Diminished lacrimal gland inflammation Protected from T1D
NOD.B10*^Tlr7-/-^*/ (52)	Systemic ablation of TLR7	* Compared to NOD.B10 age-/ sex-matched mice:* Females: Enhanced sialadenitis, total SMG B cells expanded Percentage of splenic follicular and GC B cells and ABCs decreased Reduced serum IgG titers Males: Enhanced sialadenitis, percentage of total and MZ B cells enhanced in SMG Increased pulmonary inflammation Splenic follicular B cells, ABCs, GC B cells, CD4+ activated/ memory T cells expanded Elevated serum ANA-specific IgM
NOD.B10^Tlr-DKO^/ (57)	Systemic ablation of TLR7 and TLR9	*Compared to NOD.B10 age-/ sex-matched mice:* Females: Increased pulmonary inflammation Decreased percentage of splenic B cells Reduced total and ANA-specific autoantibodies in sera Males: Reduced saliva production Decreased lacrimal inflammation Reduced total and ANA-specific autoantibodies in sera
TLR7 ko in salivary LAMP3 induction model (C57BL/6)/ (61)	Systemic ablation of TLR7 in salivary-specific LAMP3 overexpression model	*Compared to LAMP3 overexpressing age-/sex- matched mice:* Females: Protected against loss of saliva production Reduced anti-Ro and anti-La antibodies in sera
Slc29a3-/- TLR7 ko mice (C57BL/6)/ (60)	Systemic ablation of TLR7 and Slc29a3 Slc29a3-/- mice accumulate lysosomal nucleosides that activate TLR7 constitutively	*Compared to Slc29a3-/- age-/ sex-matched mice:* Females: Diminished sialadenitis (decreased B and T cells and macrophages) Decreased *CXCL9*, *CXCL13*, and *CCL5* in SMGs Males: Diminished sialadenitis (decreased macrophages)
Imiquimod (Imq) treatment (NOD.B10)/ (55)	Epicutaneous Imq administration Imq given at pre-disease time point for 6 weeks	*Compared to sham-treated NOD.B10 age-/ sex-matched mice:* Females: Cervical lymphadenopathy and splenomegaly Enhanced sialadenitis and lacrimal inflammation Elevated total and ANA-specific serum autoantibodies Expanded splenic ABCs and CD4+ activated/ memory T cells

SMG, Submandibular gland.

Indeed, treatment of pre-disease SjD females (NOD.B10Sn*H2^b^/*J, referred to as NOD.B10) with a TLR7 agonist drove robust inflammation and exacerbated both local and systemic disease manifestations and led to expansion of splenic ABCs (55). Corroborative work in NOD.B10 mice in which TLR7 was ablated systemically revealed that TLR7 altered disease severity in a sex-biased manner, as deletion of TLR7 was found to be primarily protective in females while TLR7-deficient males showed exacerbated disease (52). Additional work supports these findings, as ablation of TLR7 in females protected against salivary inflammation, loss of saliva production, and autoantibody production in other SjD mouse models (58, 60, 61).

**Figure 2:**
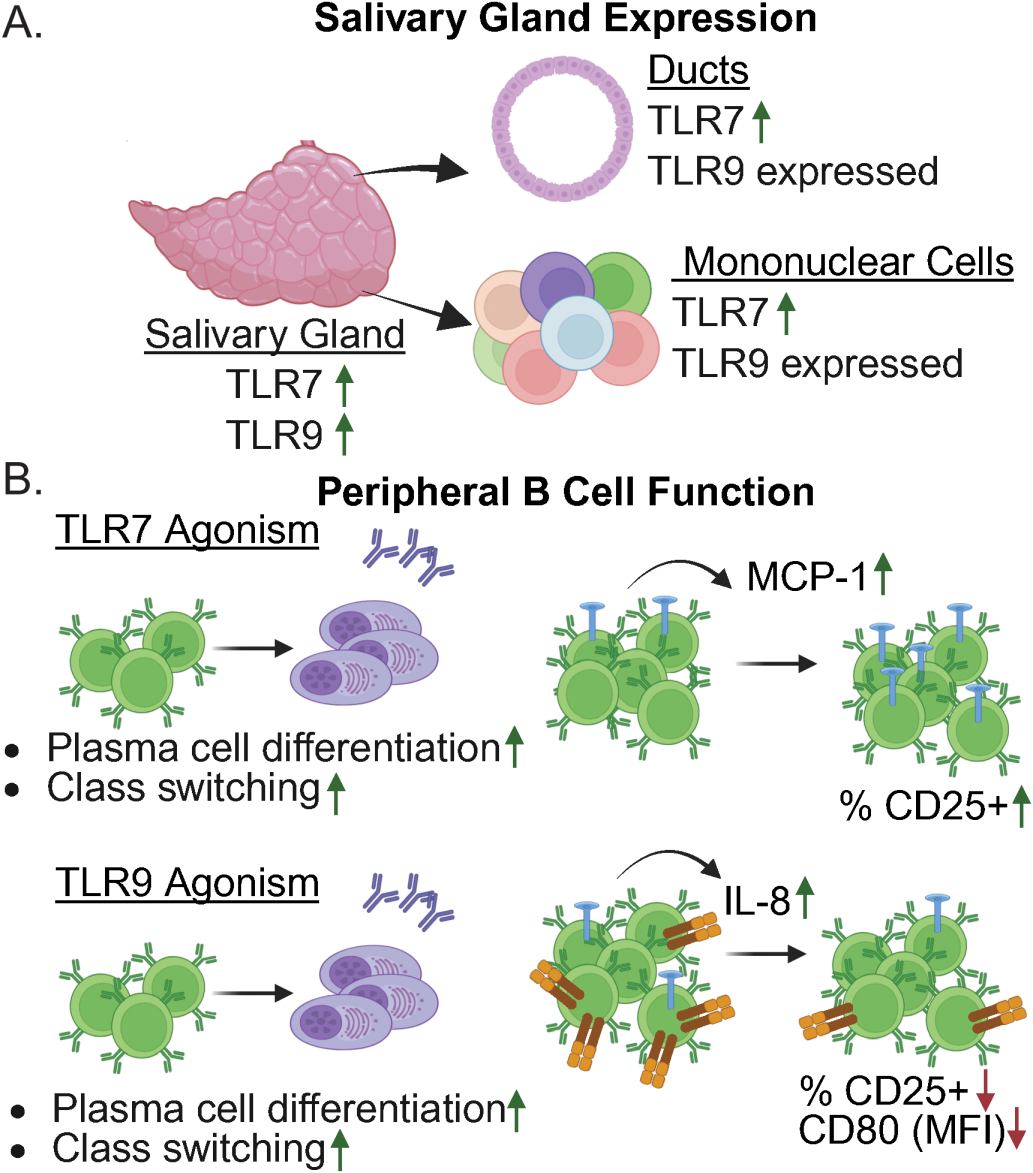
Expression and function of TLR7 and TLR9 in SjD. **(A)** Diagram summarizing expressionof TLR7 and TLR9 in salivary tissue in SjD. Relative expression compared with non-SjD controls isshown. Figure is based on (29, 49, 51, 58, 144–146) **(B)** Summary of TLR7 and TLR9 agonism of peripheral B cells in SjD. (Top panel, TLR7 agonism) shows increased plasma cell differentiation and class switching in SjD B cells compared to healthy controls (HCs) (62). Enhanced secretion of MCP-1 and an increased percentage of CD25+ B cells was observed compared with unstimulated SjD B cells (50). (Bottom panel, TLR9 agonism) shows increased plasma cell differentiation and class-switching in SjD B cells compared to HCs (62). Enhanced secretion of IL-8 was observed compared to B cells derived from HC. A decreased percentage of CD25+ B cells and reduced CD80 expression was seen compared with unstimulated SjD B cells (50). BioRender was used to create this figure.

Studies in SjD patients further support a role for TLR7 in disease. TLR7 is upregulated in salivary gland epithelial cells (SGECs), minor salivary glands (MSGs), and parotid tissue (29, 49, 51, 58, 144–146). An overview of TLR7 expression in salivary tissue from SjD patients is shown in **Figure 2A**. Data suggest that this upregulation may carry functional consequence, as TLR7 levels positively correlated with levels of proinflammatory mediators implicated in disease, such as TNFα, lymphotoxin alpha, and CXCL13 (49, 51, 58). In a separate study of MSG biopsies from SjD patients, expression of TLR7 was noted along with MyD88, TRAF6, and IRF7, suggesting activation of TLR7 in salivary tissue (145). TLR7 was also increased in PBMCs, B cells and CD14^+^ monocytes from SjD patients (51, 53, 144).

Moreover, B cells from SjD patients were hyperresponsive to TLR7 agonism, as MCP-1 secretion was enhanced in B cells from SjD patients as unstimulated unstimulated SjD B cells. This effect was specific for SjD, as there was no difference in MCP-1 production in B cells derived from healthy controls when unstimulated and TLR7-treated cells were compared (50). In addition, SjD patient-derived naïve B cells stimulated with a TLR7 agonist showed heightened plasma cell differentiation and class-switching as compared to B cells from healthy controls (62). A summary of the effects of TLR7 agonists on peripheral B cells from SjD patients is provided in **Figure 2B**. Furthermore, peripheral B cells derived from SjD patients show heightened phosphorylation of STAT3 S727 and NFκB following combined TLR7 and TLR9 stimulation, and this correlated with a type I IFN signature (64). These observations were not limited to B cells, as monocyte-derived DCs from SjD patients showed robust maturation following stimulation with a TLR7/8 agonist relative to healthy controls (147). Thus, immune cells are hyperresponsive to TLR7 ligation in the context of SjD (51, 53, 144).

Of note, similar to lupus, studies in both mice and humans indicate a role for ABCs in the pathogenesis of SjD. In the NOD.B10 mouse model, ABCs were expanded in females at the clinical disease stage (56). Moreover, studies using sort-purified splenic ABCs from SjD-prone mice revealed that this subset secreted numerous ANAs in response to TLR7 agonism, including those directed against nucleic acids (56). Interestingly, the autoantibody profile of TLR7-stimulated murine ABCs was reminiscent of that seen in SjD patient sera (56). These findings are supported by additional work using SjD patient samples, as an early study using peripheral blood revealed that ABC-like B cells (CD19+CD10-CD27-IgM+CD21^-/lo^) were expanded in SjD patients relative to healthy controls, and this population was enriched in autoreactivity towards both nuclear and cytoplasmic structures (148). Moreover, analysis of the BCR repertoire in this population identified evidence of strong selection by antigens, one of which was a ribosomal self-antigen (149). RNA sequencing studies also indicated expansion of this B cell subset in the salivary glands in disease (146, 150).

Recent work has extended these findings, as CD21^lo^ B cells were found to be expanded in peripheral blood from SjD patients, and this subset positively correlated with disease severity, as measured by ESSDAI (151). Additionally, CD21^lo^ DN B cells in the peripheral blood of SjD patients were enriched in expression of the activation marker CD86. Self-reactivity was increased in the DN subset, although this was observed in both healthy controls and SjD B cells (152). Collectively, these data indicate that TLR7 drives disease in SjD females, and sex-biased TLR7 effects may underlie the striking female predilection observed in SjD, although further studies are needed to establish this conclusively.

### 3.2 Sex- and tissue-specific contributions of TLR9 to SjD pathogenesis

Recent work examining the role of TLR9 in SjD using the NOD.B10 mouse model found that ablation of TLR9 had dichotomous effects in females and males (57). Indeed, TLR9-deficient SjD females displayed elevated percentages of splenic ABCs and GC B cells. T cell populations associated with disease were also expanded, as both splenic CD4+ and CD8+ activated/memory T cells and Tfh cells were elevated (57). Moreover, nephritis was enhanced in TLR9-/- SjD females, and anti-nuclear autoantibodies were elevated (57). In SjD males, however, ablation of TLR9 had negligible effects, with the exception of the lacrimal tissue, where inflammation was enhanced compared to the sex-matched parental strain animals (57). Thus, deficiency of TLR9 exacerbated SjD in females, while TLR9 ablation had negligible effects in males.

In SjD patients, TLR9 protein was detected in both ducts and immune cells in parotid tissue, and *TLR9* was upregulated in MSGs (51, 153) (**Figure 2A**). Of note, studies in immune populations have yielded mixed results regarding TLR9 expression in SjD. Analysis of CD14+ monocytes and PBMCs from SjD patients found decreased *TLR9* expression relative to healthy controls (54, 144), while a separate study reported increased *TLR9* expression in PBMCs (51). Functional analyses of B cells from SjD patients demonstrated enhanced secretion IL-8 following TLR9 agonism (50). Upon stimulation with the TLR9 agonist CpG, naïve B cells from SjD patients exhibited increased class-switching and plasma cell differentiation as compared to those from healthy controls (62). In good alignment with studies in lupus patients, there is evidence suggesting that B cells from SjD patients may be hyporesponsive to TLR9 agonism, as CD80 expression and the percentage of CD25+ B cells were decreased in SjD patients as compared to those from healthy controls (50). Thus, TLR9 is a potent mediator of SjD, particularly in females. Further studies are needed to determine the way in which TLR9 governs inflammation in specific cell populations in disease. A summary of the effects of TLR7 and TLR9 agonism in SjD patient B cells is provided in **Figure 2B**, and an overview of endosomal TLR expression and activation in relevant immune populations in SLE and SjD is provided in **Table 4**.

**Table 4:** SLE and SjD patients show aberrant expression and activity of endosomal TLRs in pDCs and B cells.

pDCs	SLE
	• pDCs from SLE patients had increased IFNα production when stimulated with a TLR7 agonist, but reduced IFNα production following TLR9 agonism (217–219) • BDCA2 therapy that targeted pDCs decreased expression of IFN response genes and reduced disease activity of skin disease (220–222) • PMNs release NETs that enter pDC endocytic compartments and activate them to produce high amounts of IFNα (223, 224)
	SjD
	• Type I IFN+ pDCs showed upregulation of TLR7, MyD88, and IRF7 (144) • pDCs produced higher levels of pro-inflammatory cytokines, including type-I IFN, upon stimulation with endosomal TLR ligands (225) • pDCs exhibited increased basal TLR7 expression; pDCS from SjD patients and healthy controls produced similar levels of IFNα following TLR7 or TLR9 agonism (226)
B cells (total)	SLE
	• TLR7 expression in CD19+ B cells was increased compared to healthy controls (227) • Patients with *TLR7* gain of function mutations displayed increased TLR7 expression in B cells and elevated autoantibodies (42, 43) • Patients with a specific *TLR7* SNP expressed high levels of TLR7 and had increased peripheral B cells. Overexpression of TLR7 in SLE patients drove expansion of transitional (TR) B cells. High TLR7 signaling in TR B cells promoted auto-Ab production (228) • TLR9 expression was higher in B cells from SLE patients than healthy subjects (88, 92, 229) • B cells from SLE patients were hyporesponsive to TLR9 agonism (93, 95, 96)
	SjD
	• Peripheral B cells showed increased signaling in response to TLR7/TLR9 agonism; this correlated with the type I IFN signature (64) • CD80 expression was decreased upon TLR9 agonism (50) • Naïve SjD B cells showed enhanced plasma cell differentiation and antibody secretion upon TLR7 or TLR9 agonism (62)
ABCs/DN B cells	SLE
	• DN B cells (CD19+ IgD- CD27-) were expanded in patients with SLE. This subset was hyperresponsive to TLR7 agonism (132) • A DN subset (DN3: CD19+ IgD-CD27- CD11c- CXCR5-) was associated with disease activity in females. A *TLR7* SNP was associated with altered levels of DN B cells (230)
	SjD
	• The percentage of CD19+ CD27- CD10- CD21^-/low^ B cells was expanded and enriched in autoreactivity (148) • CD19+ IgM+ CD27- CD10- CD21^-/low^ B cells showed clonal proliferation; recognition of self-antigens may drive this expansion (149) • Single cell RNA sequencing studies indicated expansion of ABC-like populations in salivary tissue from patients with SjD (146, 150)

NETs, Neutrophil extracellular traps; DN, Double negative.

### 3.3 TLR9 restrains TLR7-mediated disease manifestations in SjD females

Given the importance of TLR7 and TLR9 in SjD, further experiments were carried out to determine the disease phenotype of SjD females and males that lacked systemic expression of both TLR7 and TLR9 (57). In females, most of the SjD manifestations observed in NOD.B10*^Tlr9-/-^* mice were dependent on the expression of TLR7 (57). There were some exceptions to this observation, however, as lung inflammation was enhanced in female mice that lacked both TLR7 and TLR9 expression as compared to the parental strain. Moreover, titers of total IgM and IgG and autoantibodies were dramatically reduced in the TLR7/TLR9 double knockout mice as compared to the other female strains examined (57). In contrast, few changes were observed in SjD males lacking both TLR7 and TLR9, although the TLR7/TLR9 double knockout males showed diminished saliva production and decreased antibody titers as compared to sex-matched parental strain animals (57). Altogether, these data indicate essential roles for both TLR7 and TLR9 in SjD and provide evidence that TLR9 restrains TLR7-dependent inflammation in females in the context of SjD.

## 4 Comparison of the roles of TLR7 and TLR9 in SLE and SjD

While similarities are noted regarding the roles of TLR7 and TLR9 in SLE and SjD, it is important to point out that activation of endosomal TLRs has distinct outcomes in each disease. In this section we will review key similarities and differences between TLR7 and TLR9 in SLE and SjD.

### 4.1 TLR7 drives inflammation in females in both SLE and SjD

TLR7 is overexpressed and hyperactive in female lupus models and patients (39, 76, 154–156). Studies in conditional knockout lupus-prone females in which TLR7 was ablated only in the B cell compartment demonstrated that B cell TLR7 mediated glomerulonephritis, proteinuria, and B cell expansion (27). Moreover, female mice that expressed TLR9 containing a TLR7 TIR domain (termed TLR997) showed increased spleen weight and nephritis compared to the TLR9-sufficient parental strain (143). Altogether, these data indicate that ablation of TLR7 has profound effects on lupus manifestations in females.

In good agreement with the lupus literature (27), several disease manifestations were ameliorated in the absence of TLR7 in SjD females. Indeed, ablation of TLR7 resulted in diminished splenic B cell and reduced IgG titers in sera in SjD females (52). In corroborative studies, sialadenitis and serum autoantibodies were reduced SjD-prone females, and mice were protected from loss of saliva production (58, 60, 61). Additionally, treatment of SjD-prone females with a TLR7 agonist exacerbated disease (55). Therefore, similar to findings in lupus models and patients, TLR7 drives disease in female SjD mice.

### 4.2 TLR7 plays a pathogenic role in lupus males, but protects against disease in SjD

Similar to findings in females, many studies show that ablation of TLR7 in lupus males protects against disease. Indeed, many disease manifestations were improved in lupus males that lacked TLR7 systemically as compared to the parental strain (33, 70), and overexpression of TLR7 in lupus males resulted in disease that was reminiscent of that observed in females (66, 128) Moreover, in lupus-prone male mice that expressed TLR9 containing a TLR7 TIR domain, spleen weight and nephritis were enhanced compared to the sex-matched TLR9-sufficient parental strain (143). Altogether, these data indicate that TLR7 plays pathogenic role in lupus in males.

These findings are in contrast to those in SjD males, where murine studies report both pathogenic and protective roles for this receptor in disease. Indeed, one study found that SjD-prone males exhibited elevated inflammation in both salivary and lung tissue and had increased percentages of GC and activated memory CD4+ splenic T cells as compared to TLR7-sufficient sex-matched NOD.B10 mice (52). Work in other SjD models, however, found that ablation of TLR7 diminished salivary or lacrimal inflammation as compared to strain and sex-matched controls [**Table 3** and (59, 60)]. While further studies are needed to determine whether TLR7 is primarily protective or pathogenic, these data suggest that the role of TLR7 is distinct between males in lupus and SjD (27, 52, 143). Further studies are needed to determine whether these observations are consistent in SjD patients, as these findings have important implications for therapeutic interventions targeting TLR7 in this disease.

### 4.3 TLR9 protects against disease in lupus and SjD females

The role of TLR9 in lupus is consistent with observations made in SjD females, as many disease manifestations were exacerbated when TLR9 was ablated. TLR9-deficient lupus females showed enhanced proteinuria and glomerulonephritis (78, 157). Moreover, total IgG and anti-Sm and anti-RNA titers were elevated in sera, and splenic GC B cells were expanded in female lupus mice (78). Similarly, nephritis was enhanced in SjD females and autoantibodies were increased (57). Furthermore, splenic ABCs and GC B cells were expanded, along with CD4+ and CD8+ activated/memory T cells and Tfh cells (57). Altogether, these data indicate that TLR9 expression protects against disease in both lupus and SjD female models of disease.

### 4.4 TLR9 ablation exacerbates disease in lupus males, but has negligible effects in males with SjD

**Figure 3:**
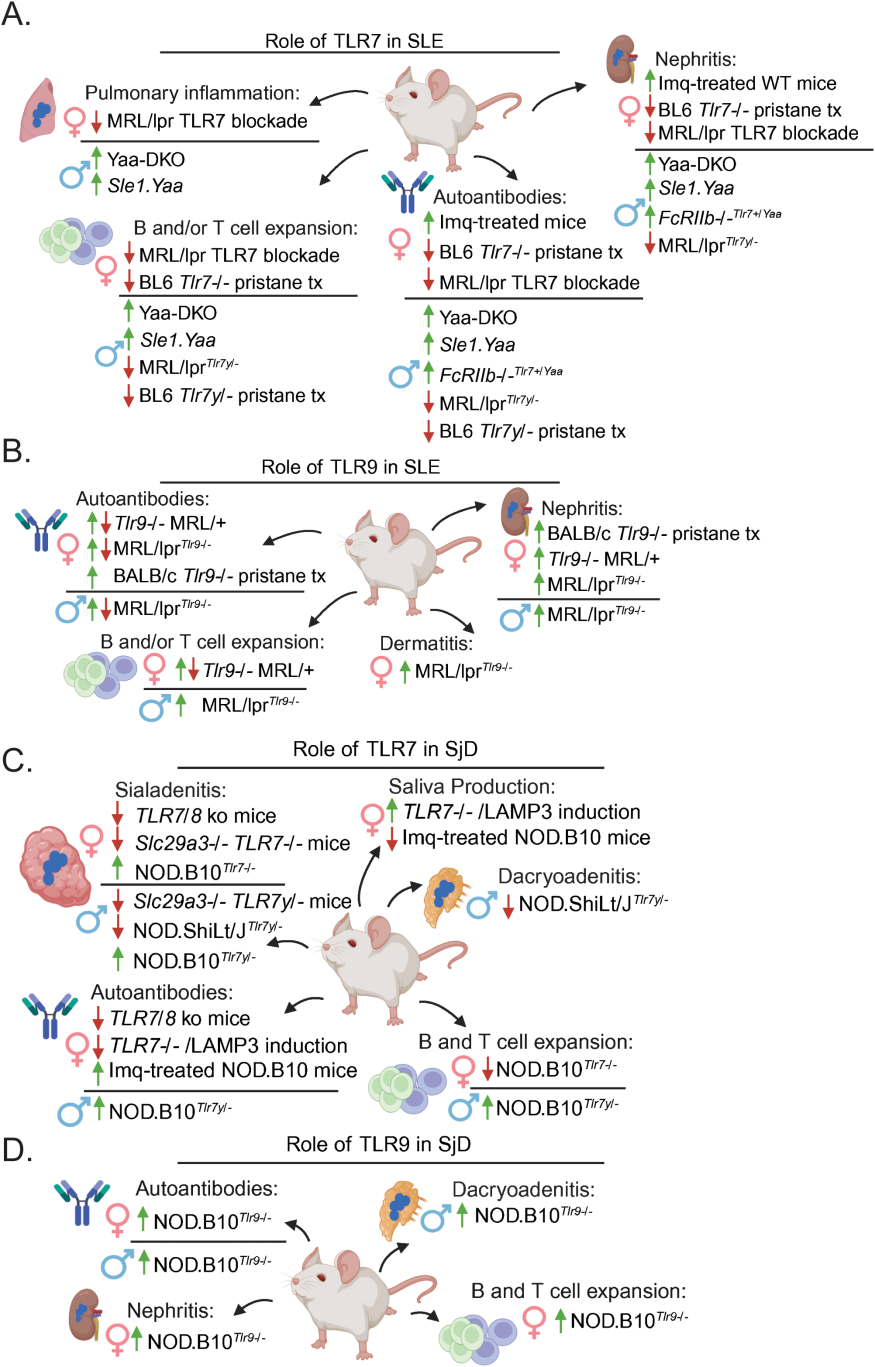
Comparison of TLR7 and TLR9-mediated sex differences in SLE and SjD. **(A)** Role of TLR7 in SLE. Figure is based on (33, 70, 72–75, 128). DKO indicates *Def6-/-* and *Swap-70-/-* model. **(B)** Role of TLR9 in SLE. Figure is based on (33, 70, 78, 79, 157). **(C)** Role of TLR7 in SjD. Figure is based on references provided in **Table 3**. **(D)** Role of TLR9 in SjD. Figure is based on (57). BioRender was used to create this figure.

Interestingly, TLR9-deficient lupus males show similar disease manifestations as those observed for TLR9-/- females. Indeed, male MRL/lpr mice that lacked systemic expression of TLR9 displayed enhanced disease, as pDC activation markers and serum IFNα levels were increased as compared to those from sex-matched parental controls (70). Moreover, serum IgG and the percentage of activated splenic B and T cells were elevated, and both kidney and skin pathology were exacerbated (70). These findings are in contrast to those in SjD, as ablation of TLR9 had little effect on organ-specific disease in NOD.B10 males (57). There were two notable exceptions to this observation, as lacrimal tissue showed enhanced inflammation in the absence of TLR9 and select histone-specific autoantibodies were also increased in male NOD.B10 mice compared to sex-matched TLR9-sufficient controls (57). Thus, in contrast with findings in lupus mice, ablation of TLR9 in SjD males had few effects on disease. A summary of TLR7 and TLR9 mediated organ-specific disease manifestations in females and males is provided in **Figure 3**.

### 4.5 TLR9 restrains TLR7-driven inflammation in females with lupus or SjD

Findings in lupus and SjD mice that lack systemic expression of both TLR7 and TLR9 are insightful in elucidating the regulatory roles of these receptors in disease. In both female and male lupus mice that lacked both TLR7 and TLR9, spleen and lymph node weight and nephritis were diminished as compared to sex- and strain-matched controls that were deficient in TLR9 (33, 143). Moreover, additional work in lupus females and males that lacked both TLR7 and TLR9 revealed that distinct autoantibodies were reduced in sera as compared to the parental strain, although there were no differences in total IgG (33). Thus, data from lupus studies revealed that in both males and females, TLR9 restrains TLR7-mediated organ-specific disease.

These findings were reminiscent of those in SjD females, as many disease manifestations in TLR9-deficient SjD females were dependent on TLR7 expression (57). In males, however, ablation of TLR7 and TLR9 resulted in disease that resembled that seen in mice that lacked TLR7 alone (52, 57). These results are consistent with data demonstrating a limited role for TLR9 in disease in SjD males.

### 4.6 Antibody production is dramatically reduced in SjD mice lacking both TLR7 and TLR9

In contrast to findings in lupus, data from SjD mice indicate that IgG production requires the dual expression of TLR7 and TLR9. Although these data are not available for lupus females, total serum IgG in MRL/lpr males that lacked both TLR7 and TLR9 was not different as compared to the parental strain, although select IgG subclasses (IgG2a and IgG3) were diminished in the double knockout males (33). These results differed significantly from those in SjD mice, where total IgG and autoantibody levels in sera were dramatically decreased in both females and males relative to sex-matched parental strain animals (57). While the reasons for these disparate findings are unclear at present, it is interesting to speculate that there may be distinct B cell-intrinsic endosomal TLR-dependent mechanisms governing IgG production in lupus and SjD. Further studies are needed to investigate the underlying reasons why TLR7 and TLR9 expression is required for IgG production in SjD and to determine whether other B cell functions are altered in the absence of both TLRs in this disease.

### 4.7 Specific endosomal TLR ligands govern inflammatory outcomes in SLE

It is important to point out that the nature of the TLR ligand is a key determinant as to whether endosomal TLR signaling is pathogenic or protective in lupus. As mentioned above, endogenous nucleic acids serve as TLR7 and TLR9 ligands in the context of autoimmunity. Under physiologic conditions, the DNAse I family, which includes DNAse I and DNAse1L3, serves to degrade extracellular nucleic acids in order to prevent their accumulation and subsequent activation of endosomal TLRs (158, 159). Mice that are deficient in either DNASE1 or DNASE1L3 develop a lupus-like disease that has many similarities to SLE patients (160, 161). Studies performed in DNASE1L3-deficient lupus-prone mice revealed that both TLR7 and TLR9 mediate disease pathogenesis, and their dual deletion resulted in suppression of autoantibody responses and amelioration of nephritis (161). These observations have important clinical relevance, as patients are identified who exhibit monogenetic null mutations in DNASE1L3, and such individuals develop SLE early in childhood. Additionally, in greater than 50% of sporadic cases of lupus with kidney disease, neutralizing autoantibodies to DNASE1L3 have been detected, which indicates that inhibition of Dnase1L3 activity by non-genetic mechanisms is a common cause of severe sporadic lupus nephritis (162, 163).

Similarly, Phospholipase D3 (PLD3) and PLD4 digest ssRNA and ssDNA, preventing subsequent activation of endosomal TLRs (162). *PDL4* was identified as an SLE risk allele, and mice lacking PDL4 developed a lupus-like disease characterized by production of ANAs and ds-DNA autoantibodies (163). Moreover, aberrant expression of PDL4 is associated with SLE in humans, as a rare population of T-bet+ PDL4+ B cells was identified in SLE patients that had overlapping features with DN B cells (164). In studies using PBMCs derived from healthy controls, TLR7 or TLR9 stimulation enhanced B cell PLD4 expression and antibodies derived from PDL4+ B cells tended to be enriched in autoreactivity (164).

Altogether, these studies indicate that both TLR7 and TLR9 may play pathogenic roles in lupus, depending on the specific ligand characteristics. Thus, it is interesting to speculate that the diminished autoantibody production observed in SjD mice that lack both of TLR7 and TLR9 may be related to the reduced ability of these mice to respond to specific endogenous nucleic acids that mediate loss of tolerance in this model through dual TLR7 and TLR9 activation (57). Further work to examine this in the context of SjD will be insightful in elucidating the roles of these receptors in this disease.

### 4.8 Comparisons of sex-specific differences between mice and humans

An important clinically-relevant question relates to whether sex-specific differences observed in SLE and SjD patients are reminiscent of those observed in mouse models. While both SLE and SjD display a strong female predominance, limited numbers of studies suggest that these diseases manifest differently in female and male patients (165–169) (**Table 5**). For example, although SLE is rare in males, renal disease, neurologic involvement, and certain hematologic parameters and autoantibodies are reported more frequently in men (168). In contrast, leukopenia and anti-Ro and -La autoantibodies were documented more frequently in women (168). This is also the case for SjD, as pulmonary disease and lymphoma were reported more commonly in men, while salivary gland hypofunction, xerostomia, and leukopenia were seen more often in women (165–167, 169).

**Table 5:** Sex-specific disease manifestations in SLE and SjD.

SLE		
Disease manifestation	More common in:	
	Females	Males
Renal disease • Diffuse proliferative nephritis, severe renal impairment		X
Skin involvement	Inconclusive findings	
Hematologic involvement		
• Hemolytic anemia		X
• Lymphopenia		X
• Thrombocytopenia		X
• Leukopenia	X	
• Low C3		X
• Reduced total complement activity		X
Autoantibodies		
• Anti-Ro	X	
• Anti-La	X	
• Anti-dsDNA		X
• Anti-Sm		X
• Anti-U1RNP		X
• Anti-cardiolipin		X
Arthritis	Inconclusive findings	
Photosensitivity	Inconclusive findings	
Neurologic involvement		X
Cardiovascular damage		X
Hepatosplenomegaly		X
SjD		
Salivary-related		
• Salivary hypofunction (objective)	X	
• Xerostomia (subjective)	X	
• Parotid enlargement		X
• Histopathology (focus score, MALT lymphoma, LELs, GCs)	Similar between sexes	
Ocular findings	Inconclusive findings	
Pulmonary disease		X
PNS involvement		X
Myositis	Similar between sexes	
Arthritis	Inconclusive findings	
Renal disease	Inconclusive findings	
High ESSDAI score		X
Hematologic involvement		
• Hypergammaglobulinemia	Inconclusive findings	
• Low complement C3 and C4	Inconclusive findings	
• Lymphocyte count		X
• Leukopenia	X	
Lymphoma		X
Autoantibodies		
• Anti-Ro	Inconclusive findings	
• Anti-La		X
• RF	Inconclusive findings	
• Anti-RNP		X
• ANA titers ≥ 1:160		X

MALT, Mucosal associated lymphoid tissue; LELs, lymphoepithelial lesions.

While these studies suggest that significant sex-biased differences in clinical manifestations may be present in both SLE and SjD, the studies involve limited numbers of patients from disparate racial and ethnic backgrounds, and thus conflicting findings are often reported. While this work provides a framework for future studies, it is difficult to compare human data with that derived from mouse models. Therefore, further work will be instrumental in determining which findings regarding sex-biased disease manifestations in mouse models are most relevant to those in humans for both SLE and SjD.

An additional question relates to whether sex-biased findings observed are model-specific. In lupus, there are numerous mouse models that have been employed to study the role of TLR7 in disease, and there is an abundance of evidence that this receptor plays a pathogenic role (170–172). There are also numerous models that have been used for the study of TLR9 in disease (170, 173), and data derived from these models indicate that overall, the results observed are consistent across the different models employed.

It is important to point out that in SjD, however, there are a limited number of models that have been generated to examine the role of endosomal TLRs in disease. Nonetheless, several models have examined TLR7 in disease, and most of these studies have indicated that TLR7 plays a pathogenic role in SjD in females. The data from male TLR7-deficient mice is less conclusive, and further studies are needed to determine whether TLR7 plays a primarily protective or pathogenic role in SjD, and/or whether this varies in an organ-specific manner (**Table 3**). Finally, TLR9 has only been studied comprehensively in the NOD.B10 model to date (**Table 3**), so additional studies in other SjD models will be instrumental in determining whether these findings extend more generally to other models of disease.

## 5 Increased TLR7 expression in females likely contributes to the sex-bias observed in SLE and SjD

Both SLE and SjD exhibit a strong female disease predominance (6). While the reasons for this are likely multifactorial, recent data suggest that dysregulated expression of TLR7 in females may contribute to this sex-bias. *TLR7* is encoded by the X chromosome, and X chromosome inactivation (XCI) is a key mechanism that governs expression of X-linked genes in females to ensure equivalent expression of X chromosome encoded genes in males and females (174). XCI is enforced by *XIST*, a long non-coding RNA that controls expression of genes expressed on the X chromosome in females (175). Studies demonstrate several immune-related genes, including *TLR7*, undergo facultative XCI escape (156). This biallelic TLR7 overexpression is documented in both B cand T cells, and *XIST* expression is shown to be essential for restraining the ABC population in SLE patients (76, 77, 174, 176). Corroborative work in mice with diminished *XIST* expression demonstrated increased expression of X-linked genes, including *TLR7*, in monocytes/macrophages, splenic DCs and B cells (177). Interestingly, female mice that expressed diminished *XIST* developed a lupus-like disease by one year of age, characterized by elevated anti-RNP-Sm and anti-DNA autoantibodies and expansion of immune subsets implicated in disease, including ABCs and GC B cells (177). Taken together, these studies indicate that increased gene dosage of TLR7 in SLE in monocytes and B cells likely contributes to the strong female disease predominance.

While XCI escape of *TLR7* has not been demonstrated in SjD patients to date, several studies indicate that this mechanism may underlie the female sex-bias. Indeed, the prevalence of SjD was increased 38-fold in patients with Klinefelter’s syndrome (47, XXY) as compared to healthy 46, XY males (178). Moreover, a separate study found that X chromosome aneuploidies were increased in patients with SjD (179). These studies suggest that X chromosome gene dosage effects contribute to disease susceptibility. While there are no studies to our knowledge that have compared TLR7 expression between males and females with SjD, one study revealed that *TLR7* was increased in peripheral B cells from SjD females as compared to sex-matched healthy controls (180). Thus, additional studies to determine the role of XCI escape in TLR7 expression are warranted to examine underlying mechanisms contributing to the female disease predominance in SjD.

Of note, since *TLR9* is not encoded by the X chromosome, it is not directly regulated by XCI. It possible, however, that other X-linked genes may be overexpressed in SLE and SjD that govern the expression and activation of TLR9-dependent signaling pathways. As discussed above, numerous studies indicate that TLR7 restrains TLR9 activation in the B cell compartment. Moreover, *TASL* (also referred to as *Cxorf21*) is an X-linked gene that escapes XCI in the context of SLE (181, 182). This is significant because both TLR7 and TLR9 rely on TASL for signal transduction (181, 183, 184), and *TASL* is increased in healthy female monocytes and B cells as compared to those from males (181). Moreover, cells derived from male and female patients with SLE showed heightened expression of *TASL* as compared to sex-matched healthy controls (181). Altogether, numerous studies demonstrate that gene dosage effects govern the expression and activation of TLR7 and TLR9 in SLE. This paradigm likely extends to SjD as well, although additional work is needed to establish this conclusively. Thus, these data indicate that XCI escape of endosomal TLR-related genes is likely a central mechanism that governs the striking sex-bias observed in both SLE and SjD.

## 6 Differences in TLR7 and TLR9 expression and signaling likely contribute to the dichotomous inflammatory effects observed

While TLR7 and TLR9 are both expressed within the endosome and show enriched expression in pDC and B cell compartments, they display distinct differences in terms of expression and activation that likely influences inflammatory outcomes in both SLE and SjD. Studies in healthy subjects demonstrated that expression of both *TLR7* and *TLR9* is very low in naïve B cells, although *TLR9* was rapidly induced following BCR crosslinking in the presence of a TLR9 agonist (185). TLR9 stimulation of naïve B cells induced their proliferation and survival and enhanced their antigen presentation ability (186). Studies in mice indicate that TLR9 activation in specific B cell subsets may help to suppress immune activation through the production of natural antibodies that diminish inflammation (187). Altogether, these data indicate that naïve B cells are able to respond rapidly to TLR9 agonists, and early engagement of TLR9 may diminish immune activation in health but could drive the expansion of pathogenic B cells in the context of autoimmunity.

In contrast to observations in naïve B cells, both TLR7 and TLR9 show high constitutive expression in CD27+ memory B cells (185). When B cells were stimulated in parallel with either a TLR7 or a TLR9 agonist, both IgM+ CD27+ memory and plasma cells were expanded and the production of ANA was enhanced (188). Of relevance to SLE and SjD, IgM+ ANA titers were elevated when B cells were incubated concomitantly with TLR7 and IFNα, as compared to those cultured with a TLR7 agonist alone (188). Of note, addition of IFNα to B cells cultured with a TLR9 agonist did not alter IgM+ ANA titers (188). These data suggest that exposure of memory B cells to type I IFNs could differentially regulate their responses in the presence of distinct TLR ligands in autoimmunity. It is important to point out that pDCs show high constitutive expression of both TLR7 and TLR9 (189, 190), the activation of which drives robust production of type I IFN, thereby enhancing the generation of autoreactive B cells (161). Thus, interactions between specific endosomal TLR-expressing B cell subsets and pDCs that are activated by endogenous nucleic acids cooperatively drive disease.

Aside from differences in expression, endosomal TLR activation is regulated by distinct mechanisms. TLR9 function is tightly controlled by UNC93B1 interactions, which govern both its trafficking and localization (191). TLR9 also requires ectodomain cleavage and ligand processing in endolysosomes in order to become activated (192–195). This requirement for ectodomain cleavage has important regulatory consequences, as TLR9 can only be activated when it is expressed within the endolysosome. In contrast, TLR7 appears to function more as a “rheostat” whereby signaling can be tightly controlled by transport of TLR7-UNC93B1 complexes into intralumenal vesicles (142). This results in termination of signaling by sequestering TLR7 from downstream signaling intermediates and may also lead to TLR7 degradation (142). Thus, differential control of TLR7 and TLR9 signaling enables precise regulation of inflammatory responses to endosomal TLR ligands and these may be dysregulated in both SLE and SjD.

Finally, elegant work in lupus models indicates that TLR9 can signal through both MyD88-dependent and -independent pathways in B cells (34). These data suggest that the MyD88-dependent arm may drive pathology, while activation of a ligand-dependent, Myd88-independent pathway may diminish inflammation (34). Altogether, these studies indicate that TLR7 and TLR9 mediate both protective and pathogenic effects in the context of autoimmunity, and these effects are highly cell type-dependent dependent and are governed by complex mechanisms that are yet to be fully elucidated.

## 7 TLR7 and TLR9 signaling pathways represent novel therapeutic targets in SLE and SjD

As indicated by the studies reviewed, modulation of endosomal nucleic acid sensing TLRs is likely to be an efficacious therapeutic approach for patients with SLE or SjD. An overview of current and emerging drugs that target TLR7 and TLR9 signaling is provided.

Hydroxychloroquine (HCQ) has been used to treat lupus for several decades. While this drug was initially developed as an anti-malarial therapy, it is also used an immunomodulatory treatment for autoimmunity (3). HCQ has several mechanisms of action (196), one of which is to prevent endosomal acidification, resulting in reduced autoantigen presentation and blockade of TLR7 and TLR9 signaling (196). HCQ is recommended for treatment of SLE patients, as this therapy improves skin and joint disease and shows beneficial immunomodulatory effects (4). HCQ has also been used with some success in SjD patients, although additional clinical trials are needed to determine whether this therapy may be more efficacious in certain subsets of SjD patients, particularly those who display a prominent B cell activation signature (197, 198). Despite the success of this approach for treatment for specific disease manifestations in both SLE and SjD, the mechanisms of action of HCQ remain incompletely understood, and patients may exhibit inadequate disease control and experience serious adverse effects (196). In particular, adverse reactions in the gastrointestinal tract are reported, such as nausea, vomiting, diarrhea and abdominal pain (199). Patients also report toxic effects of HCQ therapy that effect the central nervous system, skin, muscles, eyes, and cardiovascular system, and careful monitoring is required for patients who receive HCQ (196). Thus, additional therapeutics are needed that carry improved safety profiles, have well-defined mechanisms of action, and target specific cell populations that are implicated in disease.

Enpatoran (M5049) is a potent, selective, small molecule inhibitor of TLR7/TLR8 that blocks both synthetic ligands and natural endogenous RNA ligands (200). Studies in both the BXSB-*Yaa* and IFNα NZB/W lupus models found that enpatoran led to increased survival and significantly diminished proteinuria and inflammatory gene expression in the peripheral blood (200). Moreover, several autoantibodies were diminished in BSXB-*Yaa* mice following treatment (200). This therapy has completed phase 1b clinical trials in patients with SLE and cutaneous lupus erythematosus (CLE) and was found to be safe and well-tolerated (201). A randomized, placebo-controlled phase II study to evaluate the long-term safety and efficacy of enpatoran is currently ongoing for patients with SLE and CLE (202). This study achieved its primary endpoint, as patients receiving enpatoran exhibited improved Cutaneous Lupus Disease Area and Severity Index-Activity (CLASI-A) scores from baseline (203). CLASI is a validated outcome measure that assesses disease activity and damage in the skin (204). Additional inhibitors of TLR7/8 activation (Afimetoran) or TLR7 (SOF-SKN) are in phase I clinical trials for treatment of CLE (205, 206). These studies suggest that targeting of endosomal TLRs may be efficacious in the management of SLE, and clinical trials are warranted to evaluate this therapy in the context of SjD.

It should be noted that systemic inhibition of both TLR7 and TLR8 carries risk of adverse effects, and these should be considered in patient management. Since both TLR7 and TLR8 recognize viral-derived single-stranded RNA, it is possible that such therapies could diminish the ability of the patient to respond to viral infection (207). Moreover, vaccine efficacy could be blunted in patients taking these therapeutics, as dual TLR7/8 agonism is being tested as an adjuvant strategy to enhance the efficacy of vaccinations (208, 209). It has been demonstrated, however, that patients with inherited TLR7 deficiencies did not show impaired B cell responses to SARS-coV-2 vaccination (210), so future studies will be insightful in establishing whether this is significant concern for patients receiving these therapeutics drugs.

Additional therapeutics that target MyDDosome components, such as IRAK1 and IRAK4, are in pre-clinical development and show promise in lupus models (211–214). Collectively, these novel treatment strategies will likely benefit patients with SLE and SjD, and development of sex-specific therapeutic approaches to address distinct disease manifestations will be of significant value in mitigating inflammation driven by endosomal TLR activation in the context of autoimmunity.

## 8 Conclusion

In summary, studies in mouse models and patients demonstrate that endosomal TLRs are robust modulators of inflammation in SLE and SjD, and B cell-intrinsic TLR7 and TLR9 signals are crucial for specific disease manifestations. While emerging data indicate that these receptors have overlapping roles in both SLE and SjD in females, key differences are observed in males. While targeting of these pathways shows therapeutic promise, therapies are needed to address specific disease manifestations that are tailored in accordance with patient sex. Such approaches will lead to improved management of patients with autoimmunity governed by endosomal TLR activation.
